# Root causes of elective surgical case cancellation in Ethiopia: a systematic review and meta-analysis

**DOI:** 10.1186/s13037-020-00271-5

**Published:** 2020-12-09

**Authors:** Yeneabat Birhanu, Aklilu Endalamaw, Aynalem Adu

**Affiliations:** 1 Department of Surgical Nursing, School of Nursing, College of Medicine and Health Sciences, University of Gondar, Gondar, Ethiopia; 2 Department of Pediatrics and Child Health Nursing, School of Health Sciences, College of Medicine and Health Sciences, Bahir Dar University, Bahir Dar, Ethiopia

**Keywords:** Cancellation, Elective surgery, Ethiopia

## Abstract

**Background:**

Cancellation of elective surgical operation recognized as a major cause of emotional trauma to patients as well as their families. In Ethiopia, prevalence and root causes for elective surgical case cancellation varies from time to time in different settings. This systematic review and meta-analysis aimed to find the pooled prevalence and root causes for elective surgical case cancellation in Ethiopia.

**Methods:**

The databases for the search were Web of Science, PubMed, and Google Scholar. The last literature search was performed on February 8, 2020. To assess publication bias Egger’s regression analysis was applied. The pooled estimation was estimated using random-effects model meta-analysis. Subgroup analysis was also done based on the root causes of surgical case cancellation.

**Results:**

This meta-analysis included a total of 5 studies with 5591 study participants. The pooled prevalence of elective surgical case cancellation was 21.41% (95% CI: 12.75 to 30.06%).

Administration-related reason (34.50%) was the most common identified root cause, followed by surgeon (25.29%), medical (13.90%), and patient-related reasons (13.34%).

**Conclusion:**

The prevalence of elective surgical case cancellation was considerable. The most common root cause for elective surgical case cancellation was administration-related reasons, followed by the surgeon, medical and patient-related reasons. The causes for the surgical cancellations are potentially preventable. Thus, efforts should be made to prevent unnecessary cancellations through careful planning.

**Supplementary Information:**

The online version contains supplementary material available at 10.1186/s13037-020-00271-5.

## Background

Elective surgical case cancellation refers to a scheduled surgical procedure that not performed on a given day [1]. It has been a long-standing problem for healthcare organizations across the world [2]. Many patients could not receive elective surgery as per the schedule upon the waiting list [3].

Most hospitals invest resources to support operating suites. However, there is a concern of unanticipated cancellation of scheduled surgery [4]. In developing countries, cancellation of elective surgical operation is a common phenomenon [5].

Planned surgery cancellation is a well-recognized reflects of inefficiency in health care and/or service management [6]. It contributes to frustration and mental stress to the patients and their families [7]. It also increases the waiting of patients [8], surges the economic burden due to extended hospital stays [9, 10], and increases the risk of in-hospital death [11]. There are many reasons for the cancellation of elective surgical cases but they might differ from hospital to hospital [12]. Unexpected cancellations of planned surgery divided into avoidable and unavoidable cancellations [1]. Scheduling errors, equipment shortages, and inadequate preoperative evaluation are avoidable cancellations. Unavoidable cancellations are emergency encounters and unexpected changes in the patient’s medical status [13]. Different literature suggested that by improving the planning most cancellations are avoidable. It has also suggested that patients themselves should receive notification early about their operation day and a reminder of their appointment [14]. Involving patients in such ways may increase their satisfaction with treatment decisions during initial consultations, which is a strong predictor of attendance for surgery [15].

Based on a study in Hong Kong China, reported surgical case cancellation was 7.6% [16]. Similarly 11% in Kingdom of Saudi Arabia [17], 3.6% in Jordan [18], 1.87% in Iran [19], and 20.8% in Sub-Saharan Africa [20]. Variety of root causes listed for the cancelled operations. Of these, administrative-related accounted 30.4% [18] to 84.8% [21], patients’ related accounted 25.9% [17] to 68.28% [22], medical-related reasons and surgeon-related reasons accounted 38.2% [18], and 28% [23] respectively.

In Ethiopia, different primary studies have been conducted to determine the prevalence for elective surgical case cancellation and root causes. The proportion of elective surgical case cancellation was found in the range between 8.9 to 33.9% [24, 25] in the Ethiopian setting. Discrepancies between studies make it difficult to generalize the national estimation. Having national representative data is real to underpin effective management strategies. Thus, there is a need to estimate elective surgical case cancellation at the country level. This systematic review and meta-analysis aimed to find the pooled prevalence of elective surgical case cancellation. Besides, it explores root causes for elective surgical case cancellation in the Ethiopian setting. The review question was what are the prevalence of and root causes for elective surgical case cancellation in Ethiopia?

## Methods

### Reporting

We reported the results of this meta-analysis according to the Preferred Reporting Items for Systematic Reviews and Meta-analyses (PRISMA) guideline [26] (Additional file 1 research checklist).

### Literature search

We searched Web of Science, PubMed, and Google Scholar databases. The terms for the search were pre-defined for a comprehensive search strategy. These included all fields within records and Medical Subject Headings (MeSH terms). In the Boolean operator, within each axis, we combined keywords with the “OR” operator. Then we linked the search strategies for the two axes with the “AND” operator. The search terms used for the search were “surgical case cancellation” OR “elective surgical case cancellation” AND “prevalence” OR “magnitude” AND reasons of surgical case cancellation AND “Ethiopia”. The specific searching detail in PubMed with MeSH terms was (“magnitude of surgical case cancellation”[MeSH Terms] OR “magnitude of elective surgical case cancellation”[MeSH Terms] OR “surgical case cancellation”[MeSH Terms] OR “elective case cancellation”[MeSH Terms] AND “prevalence” [All Fields])OR “magnitude”[MeSH Terms] AND reasons of surgical case cancellation [All Fields]) AND (“Ethiopia”). The last literature search was performed on February 8, 2020. The publication year of the studies was not limited during the search.

### Study selection

We exported retrieved studies to Endnote version 7 (Thomson Reuters, London) reference manager to remove duplicated studies.

The retrieved articles were screened according to pre-defined inclusion and exclusion criteria. Discussion and/or involvement of the third reviewer resolved disagreements between two reviewers.

### Eligibility criteria

#### Inclusion criteria

Included studies were 1) articles that reported about the prevalence of elective surgical case cancellation and/or reasons for elective surgical case cancellation.2) studies published in English, and 3) studies conducted in Ethiopia before 02/08/2020. We did not limit the publication year of studies during the search.

#### Exclusion criteria

Articles available without full-text, qualitative studies, any reviews, commentaries, consultants’ corners, letters, and conference abstracts were excluded.

### Quality assessment

We used Joanna Brigg’s Institute (JBI) quality appraisal criteria [27]. It is the assessment tool used to check the quality of each article. The tool consists of nine major items. The first item is appropriate to the sample frame. The second is the appropriate sampling technique. The third is the adequacy of the sample size. The fourth is a description of the study subjects and settings. The fifth is enough coverage of data analysis. The sixth is the validity of the method for identification of the condition. The seventh item is a standard and reliable measurement for all participants. The eighth is the appropriateness of statistical analysis. And the last item is adequacy and management of response rate. Studies considered low-risk when it would fit 5 or above quality assessment checklists.

### Data extraction

Three authors extract the data. Data extracted from each article were first author, the geographical location of the study, publication year, study design, study population, sample size, the prevalence of and root causes for cancellation of elective surgery.

### Outcome measurement

This systematic review and meta-analysis have two outcomes. Firstly, to determine the prevalence of elective surgical case cancellation in Ethiopia calculated as dividing the number of elective surgical patients but whom surgical cases canceled to the total number of patients multiply by 100. A total number of patients refer to elective surgical patients in the study period. Secondly, to identify the root causes for elective surgical case cancellation.

### Data analysis

The required data were collected using a Microsoft Excel 2010 workbook form. Then, the STATA Version11 software was used to analyze the data. we used a weighted inverse variance random-effects model [28] to estimate the pooled prevalence. The I^2^ statistics was employed to assess the percentage of total variation across studies [29]. I^2^ ≤ 25% suggested more homogeneity,25% < I^2^ ≤ 75% suggested moderate heterogeneity, and I^2^ > 75% suggested high heterogeneity [29]. Egger’s regression test was also applied to assess publication bias [30]. Furthermore, we carried out the subgroup analysis based on the root causes for elective surgical case cancellation.

## Results

**Fig. 1:**
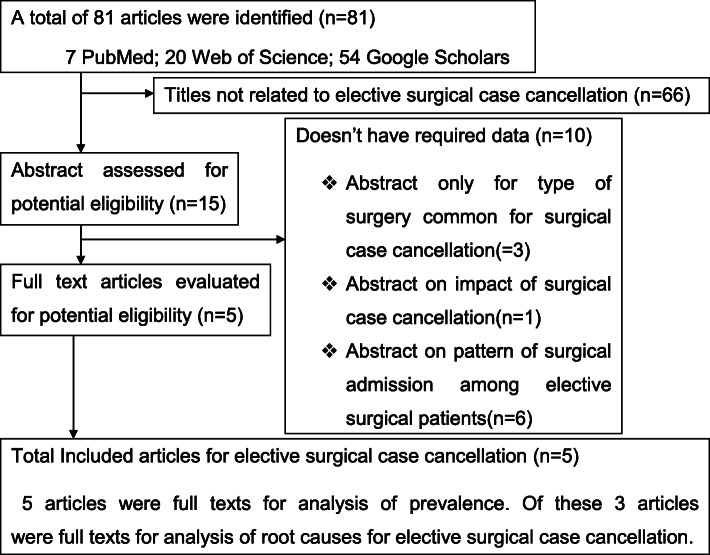
Flow chart of the literature search for the articles included in a meta-analysis of elective surgical case cancellation

A literature search of the databases yielded a total of 81 publications. Among these, 76 disregarded due to abstracts and titles that were unfit to the outcome of interest. A total of five eligible studies [24, 25, 31–33] with 5591 study participants were accessed for analysis of prevalence. Of these, three studies [24, 25, 33] with 3379 subjects were identified for analysis of root causes because the remaining two did not report about the root causes of elective surgery cancellation (Fig. 1).

### Characteristics of included studies

The range of publication year for included studies was from 2015 to 2020. We found three studies in Addis Ababa [24, 25, 31], one in Oromia [32], one in Southern Nation, Nationalities, and People Region (SNNPR) [33]. All included studies were done by using the cross-sectional study design (Table 1).

**Table 1:** Characteristics of included studies in the meta-analysis for elective surgical case cancellation

Author/Year	Study year	Region	Study design	Sample size	Prevalence	Study population
Ayele AS et al./2019 [25]	February 1 to March 1, 2016	Addis Ababa	Cross-sectional	369	33.9	All elective surgical patients
Desta M et al./2018 [33]	March 1 to 30, 2018	SNNPR	Cross-sectional	462	31.6	All elective surgical patients
Haile M and Desalegn N/2015 [32]	February 1 to June 30, 2014	Oromia	Cross-sectional	1438	23	All elective surgical patients
Bekele M et al./2020 [24]	March 1, 2018 to February 28, 2019	Addis Ababa	Cross-sectional	2548	8.9	All elective surgical patients
Gebresellassie HW and Tamerat G/2019 [31]	June 1, 2016 to May 30, 2017	Addis Ababa	Cross-sectional	774	10.7	All elective surgical patients

Three of the included studies [24, 25, 33] reported reasons for elective surgical case cancellation (Table 2).

**Table 2:** Characteristics of included studies in the meta-analysis for the root causes of elective surgical case cancellation

Author/Year	Study year	Region	Study design	Cancelled elective surgical cases	Root causes	Prevalence	Study population
Ayele AS et al./2019 [25]	February 1 to March 1, 2016	Addis Ababa	Cross-sectional	125	Patient-related reasons	13.6	All elective surgical patients
					Medical-related reasons	12	
					Administration- related reasons	30.4	
					Surgeon-related reasons	42.4	
					Emergency case priority	1.6	
Desta M et al./2018 [33]	March 1 to 30, 2018	SNNPR	Cross-sectional	146	Patient-related reasons	18.4	All elective surgical patients
					Medical-related reasons	11.6	
					Surgeon-related reasons	23.4	
					Emergency case priority	11.6	
					Administration- related reasons	21	
					Anesthesia-related reasons	14	
Bekele M et al./2020 [24]	March 1, 2018 to February 28, 2019	Addis Ababa	Cross-sectional	226	Administration- related reasons	52	All elective surgical patients
					Surgeon-related reasons	11.1	
					Emergency case priority	7.1	
					Anesthesia-related reasons	2.7	
					Medical-related reasons	17.7	
					Patient-related reasons	9.4	

### Quality assessment result

We assessed of studies with JBI quality appraisal checklists. Based on this, none of the included studies was poor quality status.

### Meta-analysis

The absence of publication bias was assessed with Egger’s regression test (*p* = 0.062), which showed that no publication bias.

**Fig. 2:**
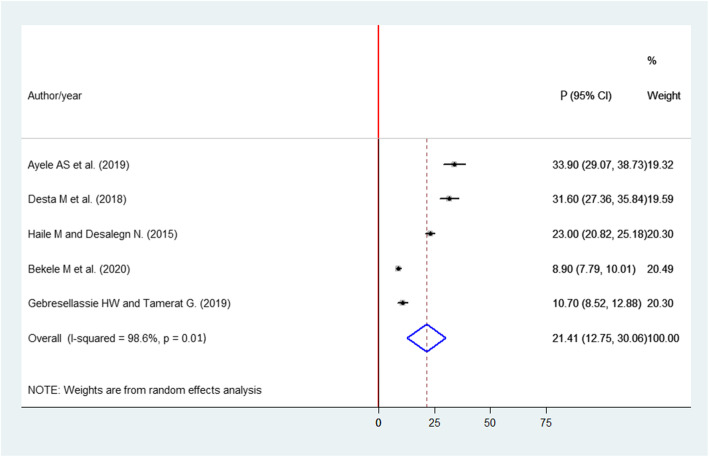
Forest plot of prevalence with corresponding 95% CIs of the five studies on elective surgical case cancellation. The midpoint and the length of each segment indicated prevalence and a 95% CI. The diamond shape showed the combined prevalence of all studies

The pooled prevalence of elective surgical case cancellation was 21.41% (95% CI 12.75 to 30.06%) (Fig. 2).

**Fig. 3:**
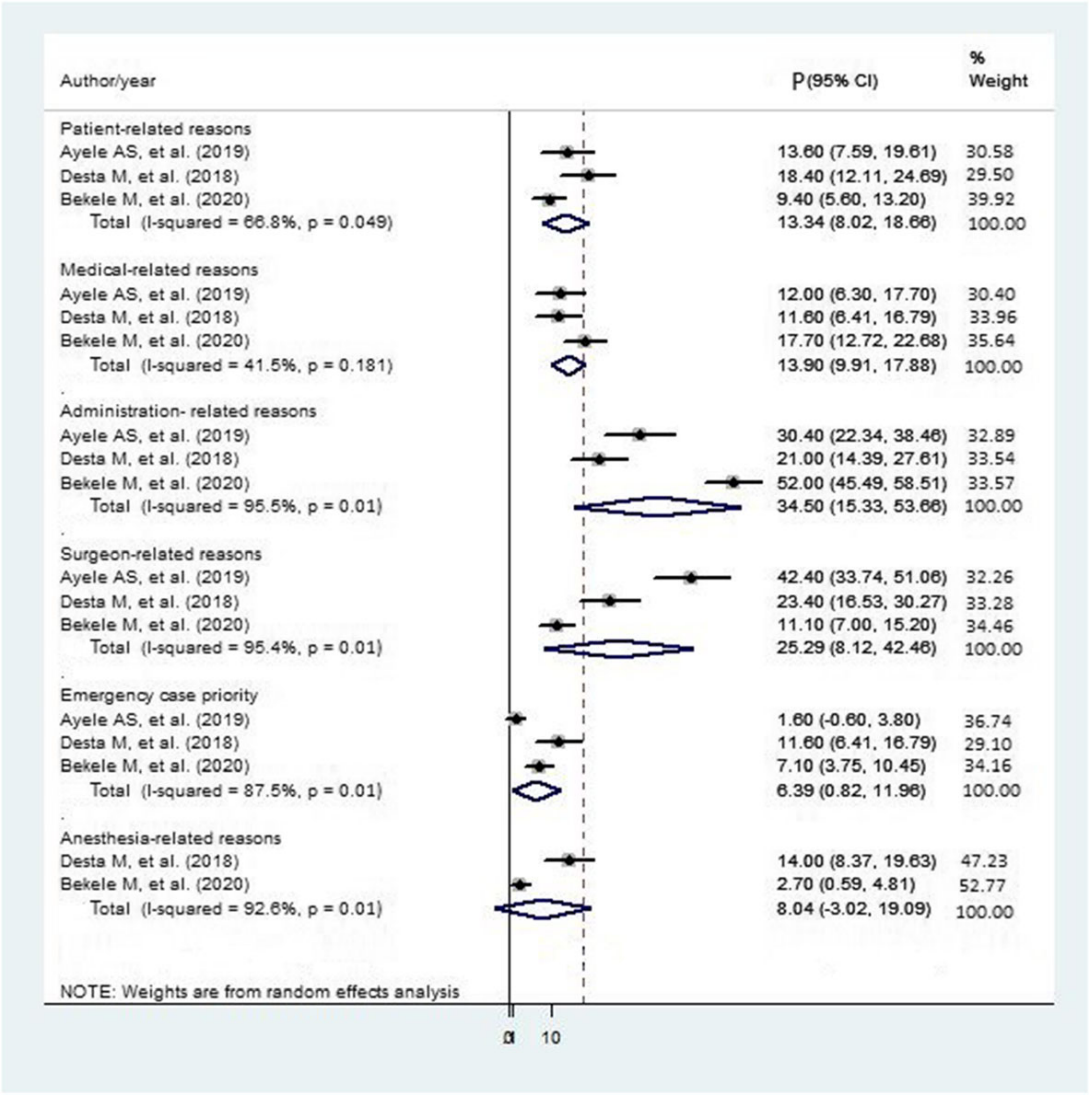
Forest plot of prevalence with corresponding 95% CIs of three studies for the root causes of elective surgical case cancellation. The midpoint and the length of each segment indicated prevalence and a 95% CI. The diamond shape showed the combined prevalence of all studies

The pooled result of root causes for cancellation of elective surgery from three studies [24, 25, 33] showed that administration-related reason (34.50%) was most prevalent followed by surgeon-related reasons (25.29%), medical-related reasons (13.90%), and patient-related reasons (13.34%) (Fig. 3).

## Discussion

There are no objective benchmarks for surgical cancellation rates. But reports under 5% are generally recommended [34]. According to this meta-analysis, the estimation of elective surgical case cancellation was 21.41% (12.75, 30.06) in Ethiopia. This is comparable with the study conducted in Sub-Saharan Africa [20] and Sudan [22]. Reasons for elective surgical case cancellation are almost similar in developing countries [20]. Besides, management strategies or surgical settings might be similar in developing countries. However, the current study’s finding is lower than a study conducted in Nigeria [23], Uganda [35] and Malawi [21]. This discrepancy might be due to findings of elective surgery cancellation vary widely because of study design; type of hospital, country, capacity, and patient type (inpatients vs. outpatients). Evidence shows that surgical case cancellation rates vary because of a lack of a standard definition, different patient populations and study methodology [36]. The current finding is higher than the study from Hong Kong China [16], the Kingdom of Saudi Arabia [17], and Jordan [18]. This difference might be due to poor hospital administration strategies. Evidence shows that lack of materials, surgeons delay, the patient not fully prepared, unperformed preliminary examinations, lack of beds in intensive care, inadequate administrative planning are indicators of poor hospital administration strategies [37]. This could cause the cancellation of elective surgical [10]. But not effective utilization of available resource hours, such as trained staff, appropriate facilities, equipment, good communication, and operational layout [38].

Based on the estimation of the root causes for elective surgical case cancellation, the most common identified cause was administration-related reason. The same report from the Kingdom of Saudi Arabia [17], Jordan [18], Uganda [35], and Malawi [21] showed that administration-related reasons found the most common causes of elective surgical case cancellation. This might be due to the reality is that surgical case cancellation can result in the financially under-utilization of theatres [2]. So, during the surgical procedure, it could cause a shortage of surgical materials in the hospital setting that makes challenge to run the activities. This finding helps healthcare policy and/or decision-makers to consider elective surgical case cancellation prevention strategies.

## Conclusion

In this finding, the prevalence of elective surgical case cancellation was considerable. The most common root causes for elective surgical case cancellation was administration-related reasons followed by surgeon-related, medical-related, and patient-related reasons. The causes for the cancellations are potentially preventable. Thus, efforts should be made to prevent unnecessary cancellations through careful planning. It means quality improvement strategies are necessary for surgical specialties that are susceptible to procedure cancellations caused by administrative reasons.

## Supplementary Information

**Additional file 1.** Research checklist.

## Data Availability

No need for more data. All information stated in the manuscript and, its supplementary information files.

## Abbreviations

CI: Confidence Interval
SNNPR: Southern Nations and Nationalities of People Region
